# Assessing Amyloid Pathology in Cognitively Normal Subjects Using ^18^F-Flutemetamol PET: Comparing Visual Reads and Quantitative Methods

**DOI:** 10.2967/jnumed.118.211532

**Published:** 2019-04

**Authors:** Lyduine E. Collij, Elles Konijnenberg, Juhan Reimand, Mara ten Kate, Anouk den Braber, Isadora Lopes Alves, Marissa Zwan, Maqsood Yaqub, Daniëlle M.E. van Assema, Alle Meije Wink, Adriaan A. Lammertsma, Philip Scheltens, Pieter Jelle Visser, Frederik Barkhof, Bart N.M. van Berckel

**Affiliations:** 1 Deptartment of Radiology and Nuclear Medicine, VU Medical Center, Amsterdam, The Netherlands; 2 Alzheimer Center and Department of Neurology, VU Medical Center, Amsterdam, The Netherlands; 3 Centre of Radiology, North Estonia Medical Centre, Tallinn, Estonia; 4 Department of Health Technologies, Tallinn University of Technology, Tallinn, Estonia; 5 Department of Biological Psychology, VU Amsterdam, The Netherlands; 6 Department of Radiology and Nuclear Medicine, Erasmus Medical Center, Rotterdam, The Netherlands; and; 7 Institute of Neurology and Healthcare Engineering, University College London, London, United Kingdom

**Keywords:** ^18^F-flutemetamol PET, amyloid pathology, visual assessment, preclinical Alzheimer disease

## Abstract

Our objective was to determine the optimal approach for assessing amyloid disease in a cognitively normal elderly population. **Methods:** Dynamic ^18^F-flutemetamol PET scans were acquired using a coffee-break protocol (a 0- to 30-min scan and a 90- to 110-min scan) on 190 cognitively normal elderly individuals (mean age, 70.4 y; 60% female). Parametric images were generated from SUV ratio (SUVr) and nondisplaceable binding potential (BP_ND_) methods, with cerebellar gray matter as a reference region, and were visually assessed by 3 trained readers. Interreader agreement was calculated using κ-statistics, and semiquantitative values were obtained. Global cutoffs were calculated for both SUVr and BP_ND_ using a receiver-operating-characteristic analysis and the Youden index. Visual assessment was related to semiquantitative classifications. **Results:** Interreader agreement in visual assessment was moderate for SUVr (κ = 0.57) and good for BP_ND_ images (κ = 0.77). There was discordance between readers for 35 cases (18%) using SUVr and for 15 cases (8%) using BP_ND_, with 9 overlapping cases. For the total cohort, the mean (±SD) SUVr and BP_ND_ were 1.33 (±0.21) and 0.16 (±0.12), respectively. Most of the 35 cases (91%) for which SUVr image assessment was discordant between readers were classified as negative based on semiquantitative measurements. **Conclusion:** The use of parametric BP_ND_ images for visual assessment of ^18^F-flutemetamol in a population with low amyloid burden improves interreader agreement. Implementing semiquantification in addition to visual assessment of SUVr images can reduce false-positive classification in this population.

Alzheimer disease (AD) is the most common cause of dementia, accounting for 60%–80% of cases above 65 y of age (1). Its pathologic hallmark is the accumulation of the amyloid-β peptide, thought to start years before cognitive impairment (2). In fact, abnormal amyloid-β levels are seen in 20%–40% of cognitively normal subjects between the ages of 60 and 90 y (3). These subjects are considered to be in the preclinical stage of AD (4,5), which provides a unique opportunity for secondary prevention studies and is gaining increasing research focus (6). To this end, reliable identification of amyloid pathology in vivo using PET is of the utmost importance in this population.

The identification of amyloid burden by means of visual interpretation of summed late images or of semiquantitative SUV ratio (SUVr) images is currently suggested to be sufficient. Previous studies have shown a high interreader agreement for the visual assessment of SUVr images and a high imaging–pathology correlation in clinical populations and end-of-life subjects (7–9). It has been shown, however, that SUVr overestimates amyloid burden compared with quantitative nondisplaceable binding potential (BP_ND_) (10). As such, quantitative BP_ND_ images may be more reliable also for visual interpretation. In a memory clinic population, Zwan et al. showed that visual assessment of parametric BP_ND_^11^C-Pittsburgh compound B images resulted in a higher interreader agreement than the frequently used SUV and SUVr images (11). To date, it remains to be determined whether these findings translate to the increasingly available ^18^F-labeled amyloid-β targeting tracers, such as ^18^F-flutemetamol, and, more importantly, to the challenging population of cognitively normal elderly participants who generally have a minimal amyloid load.

The purpose of this study was to compare 2 parametric imaging methods (SUVr vs. BP_ND_) to determine the optimal approach for assessment of early amyloid pathology. To this end, we investigated the agreement in visual assessment of SUVr and BP_ND_ images between 3 readers and its relationship to (semi-)quantitative measures.

## MATERIALS AND METHODS

### Project

The data used in this study originate from the Innovative Medicines Initiative of the European Medical Information Framework for AD (http://www.emif.eu/). The overall aim of this project is to discover and validate diagnostic markers, prognostic markers, and risk factors for AD in nondemented subjects.

### Subjects

In total, 199 subjects from the preclinical AD cohort were included at the Vrije Universiteit (VU) Medical Center. Inclusion criteria were an age of at least 60 y and normal cognition according to a delayed recall score that was more than −1.5 SDs of the demographically adjusted normative data on the Consortium to Establish a Registry for Alzheimer Disease 10-word list (12), a Telephone Interview for Cognitive Status–modified score of 23 or higher (13), a 15-item Geriatric Depression Scale score of less than 11 (14), and a Clinical Dementia Rating Scale score of 0 (15). Exclusion criteria were any physical, neurologic, or psychiatric condition that interferes with normal cognition. PET acquisition failed in 3 subjects, and 6 BP_ND_ images were lacking a visual assessment, resulting in 190 subjects who had a visual assessment for both SUVr and BP_ND_ images. PET quantification failed in 5 subjects; thus, 185 subjects were used for the quantitative analysis. Written informed consent was obtained from all subjects, and the study was approved by the Medical Ethics Review Committee of the VU University Medical Center.

### PET

PET scans were obtained using an Ingenuity TF PET/MRI camera (Philips Healthcare). Thirty-minute scans were acquired immediately after a manual injection of ^18^F-flutemetamol (191 ± 20 MBq) (16). After 60 min, during which the patient remained outside the scanner bed, a second scan of 20 min was acquired, starting 90 min after injection (17). Immediately before each part of the PET scan, a T1-weighted gradient echo pulse MRI scan was acquired for attenuation correction of the PET data. The first emission scan was reconstructed into 18 frames of increasing length (6 × 5, 3 × 10, 4 × 60, 2 × 150, 2 × 300, and 1 × 600 s) using the standard line-of-response–based row-action maximum-likelihood algorithm for the brain. The second scan was reconstructed with the same algorithm into 4 frames of 300 s each. First, Vinci Software, version 2.56 (Max Planck Institute for Neurologic Research), was used to combine the 2 PET scans into a single multiframe image. Next, each individual’s T1-weighted MR images were coregistered to the dynamic PET images using the generic multimodality setting of Vinci with a linear rigid-body schema and normalized mutual information as the similarity measure. Parametric BP_ND_ images were generated from the entire image set using the receptor parametric mapping implementation in PPET (18–20). Generation of the SUVr images was based on the 90- to 110-min scan interval. Next, T1-based volumes of interest using the Hammers atlas implemented in PVElab software were projected onto the PET images to extract regional values (21). Cerebellar gray matter was used as reference tissue for both analyses (22). Finally, we computed global values based on the average of frontal (volume-weighted average of superior, middle, and inferior frontal gyrus), parietal (volume-weighted average of posterior cingulate, superior parietal gyrus, postcentral gyrus, and inferolateral remainder of parietal lobe), and temporal (volume-weighted average of parahippocampal gyrus; hippocampus; medial temporal lobe; and superior, middle, and inferior temporal gyrus) regions (23,24).

### MRI

Whole-brain scans were obtained using the 3-T Achieva scanner (Philips Healthcare) of the PET/MRI system described above equipped with an 8-channel head coil. Isotropic structural 3-dimensional T1-weighted images were acquired using a sagittal turbo field echo sequence with the following settings: 1.00 × 1.00 × 1.00 mm voxels, repetition time of 7.9 ms, echo time of 4.5 ms, and flip angle of 8°. A 3-dimensional sagittal fat-saturated fluid-attenuated inversion recovery sequence was acquired using the following settings: 1.12 × 1.12 × 1.12 mm voxels, repetition time of 4,800 ms, echo time of 279 ms, and inversion time of 1,650 ms. The structural 3-dimensional T1 and 3-dimensional fluid-attenuated inversion recovery images were used for assessment of global cortical atrophy (25), average medial temporal atrophy (26), and Fazekas score for white matter hyperintensities (27,28).

### Visual Assessment of PET Images

Three trained readers, masked to clinical information, first assessed all SUVr images and subsequently all BP_ND_ images, in a randomized order. Images deemed dubious by the reader were reassessed on a separate occasion. Images were scaled to 90% of the pons signal using rainbow color scaling, and transverse, sagittal, and coronal views were displayed using the software package Vinci, version 2.56. Images were rated as either positive (binding in one or more cortical brain regions or striatum unilaterally) or negative (predominantly white matter uptake) according to criteria defined by the manufacturer (GE Healthcare). PET images were assessed together with a T1-weighted MR scan to limit the influence of atrophy on the visual assessment.

The level of experience in visual assessment of ^18^F-flutemetamol images differed among readers: a nuclear medicine physician with considerable experience, a nuclear medicine physician trainee with basic experience, and a radiologist in training with 6 mo of experience in nuclear medicine. All readers completed the ^18^F-flutemetamol reader training provided by GE Healthcare.

### Statistical Analysis

Baseline demographics were assessed using simple descriptive statistical analyses. κ-statistics were used to asses interreader agreement among the 3 readers, intrareader agreement between the 2 methods, and agreement between visual and semiquantitative classifications. Agreement was considered poor if κ was less than 0.20, satisfactory if κ was 0.21–0.40, moderate if κ was 0.41– 0.60, good if κ was 0.61–0.80, and excellent if κ was more than 0.80. Differences in MRI measurements between PET-negative and PET-positive cases were assessed using a Mann–Whitney *U* analysis. The correlation between semiquantitative SUVr and BP_ND_ measurements was assessed using Spearman ρ. Cutoffs were calculated for both SUVr and BP_ND_ using a receiver-operating-characteristic analysis and the Youden index. Possible overestimation of amyloid burden using semiquantitative SUVr was investigated by calculating the difference between SUVr − 1 and BP_ND_ values. Differences in global overestimation between PET-negative and PET-positive cases were assessed using a Mann–Whitney *U* analysis. Regional differences in binding and overestimation were assessed using a Wilcoxon paired test. Amyloid status resulting from quantitative assessment was considered the true amyloid status for all analyses, in the absence of postmortem confirmation.

## RESULTS

Baseline demographics are provided in Table 1.

**TABLE 1:** Demographics, MRI Measurements, and PET Values

Parameter	Data
Total cohort (*n*)	190
Women (*n*)	113 (59.5%)
Age (y)	70.4 ± 7.56
MMSE score	29 ± 1.13
Education (y)	15.15 ± 4.42
Global cortical atrophy score (0–3)	0.79 ± 0.72
Medial temporal atrophy score (0–4)	0.65 ± 0.72
Fazekas score (0–3)*	1.18 ± 0.82
Quantitative cohort (*n*)	185
SUVr	
Mean ± SD	1.33 ± 0.21
Range	0.79–2.13
BP_ND_	
Mean ± SD	0.16 ± 0.12
Range	0.20–0.66
Concordant cohort (*n*)	149
PET-negative (*n*)	130
Global cortical atrophy score (0–3)	0.74 ± 0.67
Medial temporal atrophy score (0–4)	0.57 ± 0.64
Fazekas score (0–3)*	1.18 ± 0.83
SUVr	
Mean ± SD	1.25 ± 0.09
Range	1.08–1.63
BP_ND_	
Mean ± SD	0.12 ± 0.05
Range	0.02–0.27
PET-positive (*n*)	19
Global cortical atrophy score (0–3)	0.89 ± 0.81
Medial temporal atrophy score (0–4)	0.82 ± 0.75
Fazekas score (0–3)*	1.26 ± 0.87
SUVr	
Mean ± SD	1.83 ± 0.16^§^
Range	1.54–2.13
BP_ND_	
Mean ± SD	0.43 ± 0.12^§^
Range	0.27–0.66

* White matter hyperintensity (0 = 35; 1 = 101; 2 = 40; 3 = 14).

§ *P* < 0.01, compared with PET-negative group.

### Visual Reads

**FIGURE 1.**
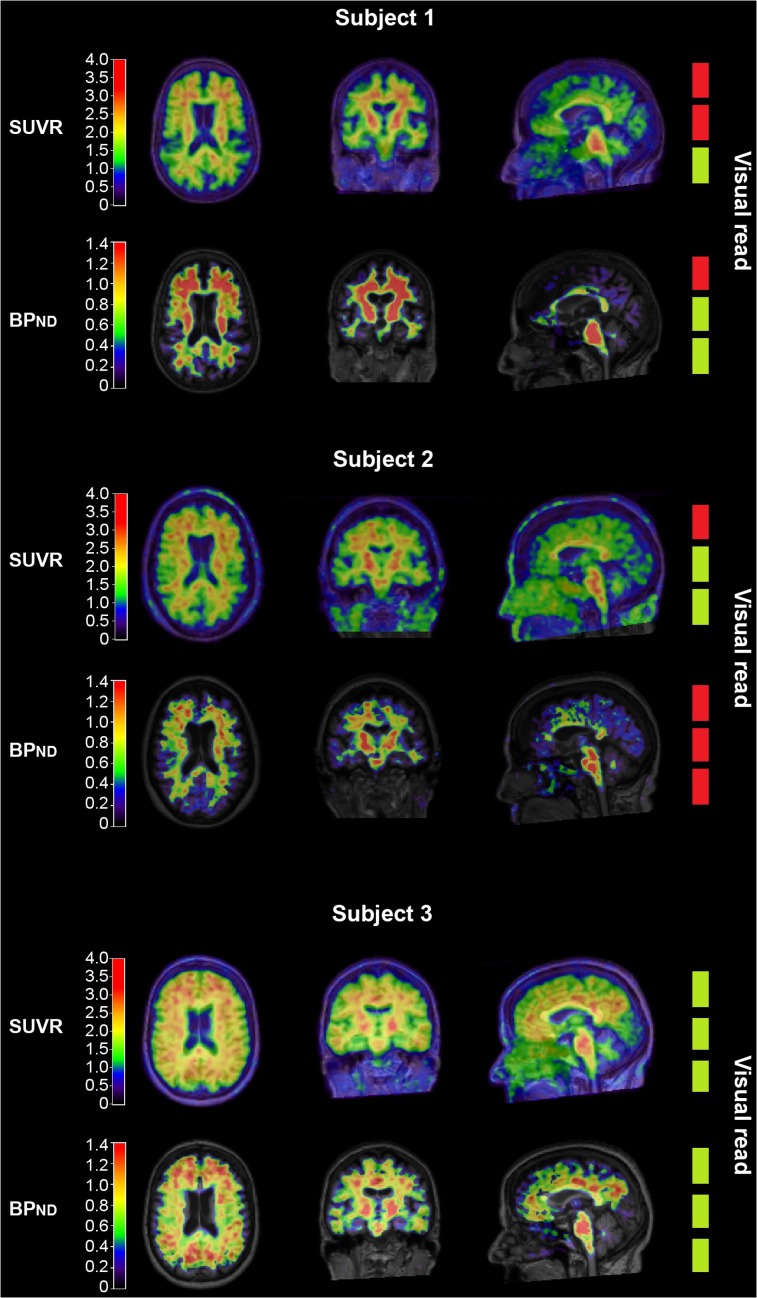
Examples of SUVr and BP_ND_^18^F-flutemetamol images of 3 different patients. From left to right are shown axial, coronal, and sagittal views. The 3 boxes on right represent amyloid classification by 3 readers (red = negative; green = positive). Subject 1: Example of difficult case, represented by discordant visual reads on both SUVr and BP_ND_ image. Subject 2: Example of possible overestimation of amyloid pathology when only SUVr image is assessed. Subject 3: Example of clearly positive case.

Interreader agreement in visual assessment was moderate for SUVr images (κ = 0.57) and good for BP_ND_ images (κ = 0.77). There was discordance between readers for 35 cases (18%) using SUVr and for 15 cases (8%) using BP_ND_, with 9 overlapping cases. Figure 1 shows examples of agreement and disagreement in visual interpretation of ^18^F-flutemetamol images. On average, the rating was positive in 35 (18%) of the SUVr images and in 26 (14%) of the BP_ND_ images. The reader with the least experience classified 55 (29%) SUVr images as positive, compared with 21 (11%) and 29 (15%) by the intermediate and most experienced readers, respectively.

Intrareader agreement (i.e., within reader, between SUVr and BP_ND_) differed among readers, with moderate agreement (κ = 0.52) between methods seen in the reader with least experience, excellent agreement (κ = 0.97) in the reader with moderate experience, and good agreement in the reader with most experience (κ = 0.76).

**FIGURE 2.**
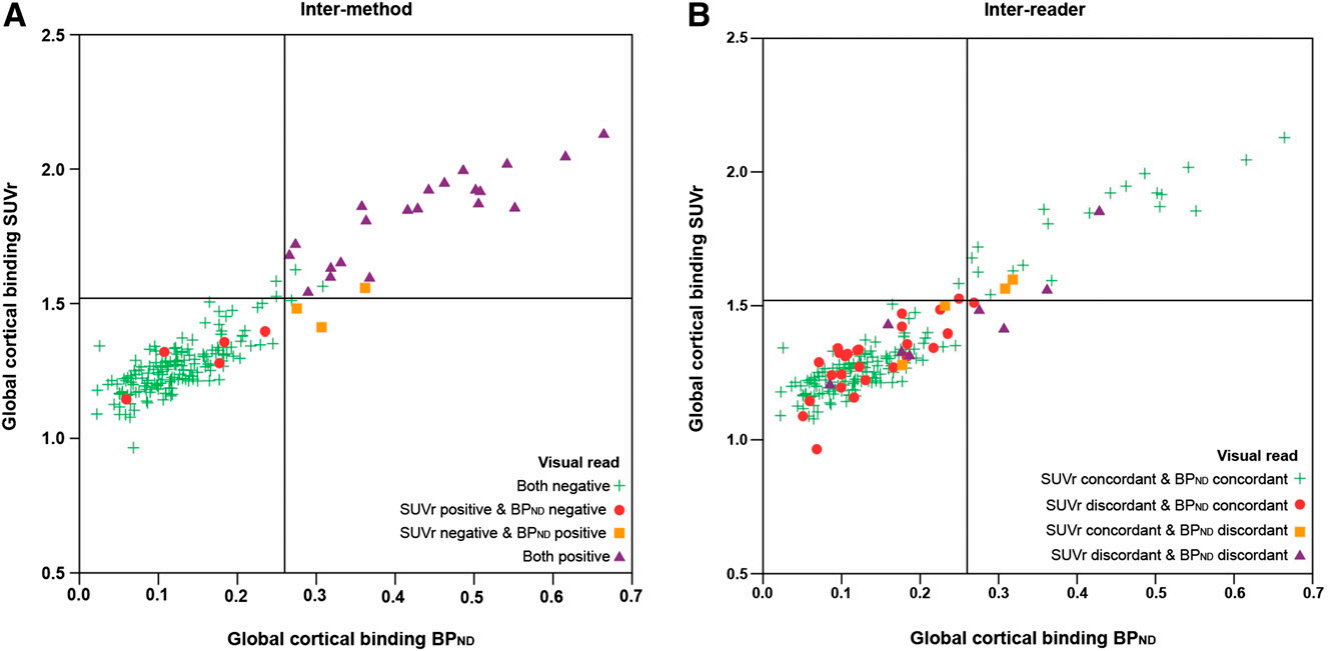
Scatterplot of quantitative measures compared with visual read. On *x*-axis is global cortical binding derived from BP_ND_. On *y*-axis is global cortical binding derived from SUVr. Reference lines denote cutoff (1.52 for SUVr and 0.26 for BP_ND_). Different colors demonstrate discordance or concordance between SUVr and BP_ND_ visual read. (A) Visual read based on majority rules. For all intermethod discordant cases (red circles and orange squares), BP_ND_ visual read was in accordance with quantitative value, whereas SUVr was not. (B) Most SUVr interreader discordant cases (red circles) are below cutoff for both SUVr and BP_ND_.

When applying majority rules (i.e., 2 of 3 readers agreed on a scan being either positive or negative), positivity was assigned to 27 (14%) cases based on SUVr and to 25 (13%) cases based on BP_ND_, with 22 overlapping cases. Thus, 8 cases showed intermethod discordance; that is, 5 cases were rated positive on SUVr but negative on BP_ND_, and 3 cases were rated positive on BP_ND_ but negative on SUVr. The remaining 160 cases were classified as negative on both images (Fig. 2A).

### Visual Reads Related to Quantitative Measures

For the total cohort, mean global SUVr and BP_ND_ were 1.33 ± 0.21 and 0.16 *±* 0.12, respectively. There was good agreement between both measures (intraclass correlation coefficient, 0.89; *P* < 0.01). Interreader-concordant positive cases had significantly higher SUVr and BP_ND_ than concordant negative cases (*P* < 0.01) (Table 1). Based on the visual read–concordant cohort alone (*n* = 149), the cutoff for positivity was 1.52 for SUVr (area under the curve, 0.98; sensitivity, 95%; specificity, 98%) and 0.26 for BP_ND_ (area under the curve, 1.00; sensitivity, 100%; specificity, 98%) using a receiver-operating-characteristic analysis (Supplemental Fig. 1; supplemental materials are available at http://jnm.snmjournals.org). After applying both cutoffs to the dataset, the agreement between the SUVr majority visual read and semiquantitative negative–positive classification was good (κ = 0.78), with 16 cases (9%) discordant between the 2 classification methods. The agreement analysis was also done with a literature-based cutoff (1.56) (8,29) resulting in a κ increase of 0.01. For BP_ND_, the agreement between the majority visual read and the quantitative negative–positive classification was excellent (κ = 0.93), with 3 cases (2%) discordant between the 2 classification methods. Most of the 35 cases (91%) for which SUVr image assessment was discordant between readers were classified as negative using either cutoff (Fig. 2B). In addition, in the 8 cases with a discordant intermethod visual read, there was full agreement between visual and quantitative measurements when BP_ND_ was used, which was not the case with SUVr (Fig. 2A).

### SUVr ≠ BP_ND_ Quantification

**FIGURE 3.**
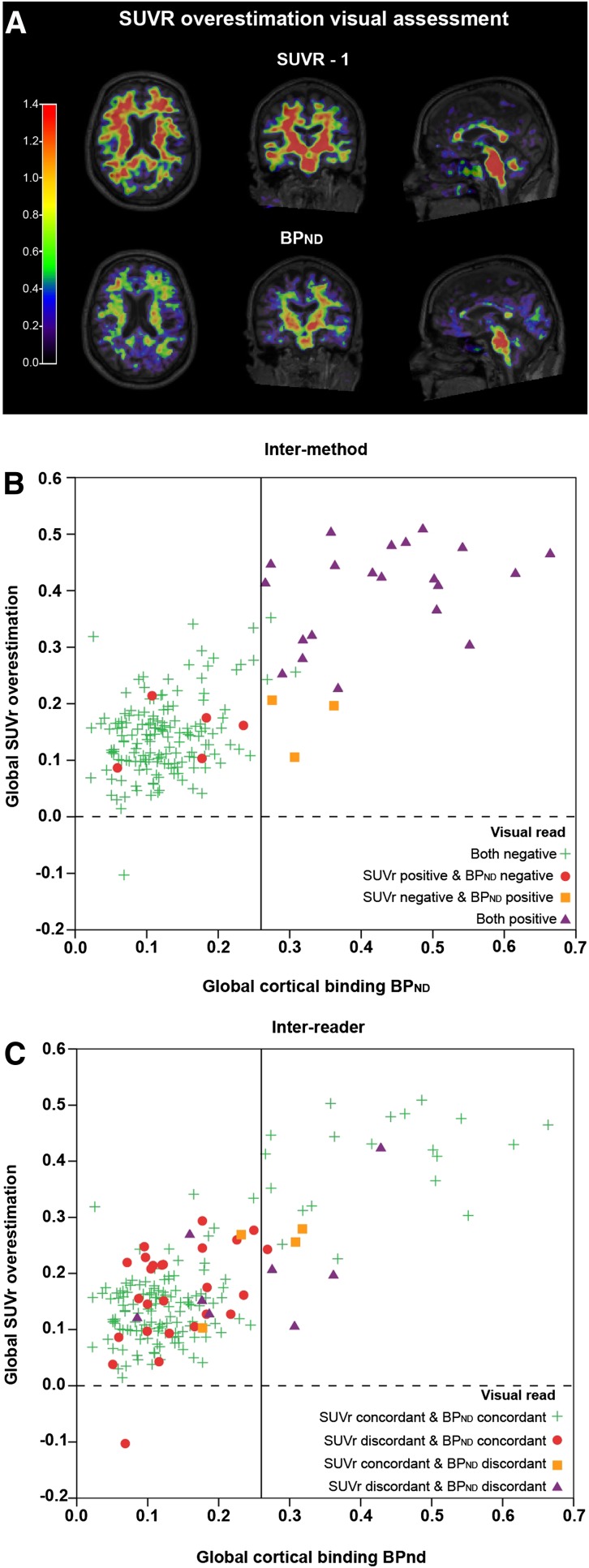
Illustration of binding overestimation for semiquantitative PET acquisition. (A) From left to right are shown axial, coronal, and sagittal views. SUVr images with subtraction of 1 show clearly higher binding values than BP_ND_ images, whereas in theory these images should be same. (B and C) Diagrams showing difference between SUVr − 1 and BP_ND_ for each subject with regard to visual read. Overestimation of SUVr is higher with increasing cortical binding.

We investigated the relationship between the 2 quantitative measures with regard to the majority visual read to assess any violations of the equilibrium assumptions (i.e., SUVr – 1 = BP_ND_) in this population. For all cases except one, global SUVr – 1 values overestimated the corresponding global BP_ND_ values. Participants with a positive read (mean overestimation [difference SUVR − 1 and BP_ND_] = 0.37 ± 0.11) had a significantly higher overestimation than participants with a negative read (mean overestimation = 0.14 ± 0.07; *P* < 0.01). This relationship was also observed on a regional level, with the frontal lobe displaying the highest mean binding and the largest mean SUVr overestimation, compared with the parietal (*P* < 0.01) and temporal (*P* < 0.01) lobes. In turn, the parietal lobe did not show a significantly higher mean binding (*P* = 0.1) but did show a significantly larger overestimation (*P* < 0.01) than the temporal lobe (Supplemental Fig. 2; Table 1). The SUVr overestimation seems to have a limited influence on the visual read of the high-binding group (i.e., BP_ND_ > 0.26), considering no cases were visually assessed as positive on SUVr and negative on BP_ND_ and only 2 SUVr images (7%) had a discordant read. For the low-binding group (i.e., BP_ND_ ≤ 0.26), the SUVr overestimation might have influenced the visual read, considering that 26 cases (16%) had a SUVr-discordant visual read. However, no obvious pattern was discernible (Fig. 3).

## DISCUSSION

In a cognitively normal elderly population with low amyloid burden, we show a considerable improvement in interreader agreement of ^18^F-flutemetamol visual assessment when using BP_ND_ rather than standard SUVr images. Misclassifications can be reduced using semiquantitative SUVr measures and avoided using fully quantitative BP_ND_ measures.

Our results are in line with the ^11^C-Pittsburgh compound B findings of Zwan et al., who found a comparable improvement in interreader agreement using BP_ND_ images (11). This result suggests that the underlying reason for discrepant interreader agreements was tracer-independent and likely related to the distinctive metrics being used (SUVr and BP_ND_). SUVr is commonly used as a proxy for BP_ND_, under the assumption that a secular equilibrium is reached during scanning. However, these equilibrium conditions are rarely met in practice. As such, whereas parametric BP_ND_ images reflect the density of available receptors (amyloid plaques), SUVr images are affected by a nondisplaceable (free and nonspecific) signal and may be affected by changes in regional flow and washout effects (28,30). As a result, SUVr can overestimate specific binding (10) and influence visual assessments (Fig. 3). Furthermore, our existing data show that this overestimation is not constant but instead increases with higher tracer binding (10,28).

The interreader agreement for the SUVr images and the concordance between semiquantitative and corresponding visual read classifications in our study are lower than previously reported (7–9). However, previous results were based on a clinical population of end-of-life subjects with a higher incidence of moderate to severe amyloid burden, which highlights the challenge of assessing amyloid pathology in a population with low amyloid burden. The challenge could be due to the nonspecific white matter uptake seen with ^18^F-flutemetamol, which together with the overestimation resulting from static scanning may translate into a tendency to visually assign regions as positive (31). In our study, the frontal regions were most often perceived as difficult to assess, leading to the greatest doubt for final classification. Although the ^18^F-flutemetamol reader training focuses on disentangling the white matter pattern from the cortical signal, assessment in this population seems additionally challenging, especially for less experienced readers. Indeed, the positive-assigning tendency was the strongest for the reader with the least experience, who also showed the lowest intrareader agreement between methods. This result stresses the need for experienced readers to make early assessments or for the reading guidelines to be updated, with the focus being on a cognitively normal elderly population. Of note, whereas the reference region used for visual assessment (i.e., pons) is different from that used for quantitative assessment (i.e., gray matter cerebellum), a separate agreement analysis using pons for quantification did not affect the agreement between classification methods.

Our results may have consequences for drug-intervention studies focused on early populations, since using the visual assessment of SUVr images as an inclusion criterion could result in false-positive inclusion due to the observed overestimation of cortical amyloid burden (32,33). Also, studies indicate that cerebral blood flow can change with age and disease progression (34,35). Therefore, using BP_ND_ images in clinical trials could avoid false-positive classification in visual assessment (28) and ensure that measured changes are due to the treatment instead of a measurement error or blood flow confounders.

An important factor in considering dynamic PET acquisition is participant burden. In this cohort, 95% of participants indicated they had no objections to undergoing a second dynamic PET scan. The coffee-break protocol used in this study may have facilitated this response and suggests the feasibility of longitudinal dynamic acquisition in cognitively normal elderly persons.

In a clinical setting, however, amyloid burden will more likely be moderate to severe and dynamic acquisition more challenging. In addition, the utility of SUV or SUVr visual reads for the diagnosis of AD-type dementia in a clinical setting has been extensively shown (36). Thus, in this context, visual assessment of SUVr images may indeed be sufficient. Nevertheless, the present results illustrate that semiquantification using SUVr can help reduce false-positive classification, especially in a challenging population. Thus, the clinical preference for visual assessment could be revised in light of more available automatic semiquantification methods, such as the one already provided for ^18^F-flutemetamol PET scans (8).

In this study, the standard manufacturer guidelines were used for reading both SUVr and BP_ND_ images. Nonetheless, an interesting finding was the improvement in interreader agreement for BP_ND_ images despite the lack of official guidelines and the limited experience of readers in assessing such images. However, it might still be of interest to formally assess whether the current guidelines are optimal for assessing BP_ND_ images. In addition, optimizing visual assessment of SUVr images by updating the current guidelines and providing training specifically focused on early accumulation may also improve the certainty of classification, comparable to that observed using dynamically derived measures. Studies have suggested that, specifically, medial frontal, anterior/posterior/isthmus cingulate cortex, and precuneus are early-accumulating regions (37,38). These regions can be visually assessed using the sagittal view of the PET image. Thus, the importance of this plane may be of most interest for updating guidelines.

A limitation of this study is the lack of a gold standard, as no postmortem data were available, hampering the understanding of the findings in relation to underlying neuropathology. Furthermore, although the frequency of amyloid positivity in this cohort is comparable to previous reports (39), the low incidence may have induced reader bias with regard to searching for amyloid positivity. Lastly, both quantification and visual assessment of the PET images in this study were accompanied by structural MRI, which might not always be available.

## CONCLUSION

The use of parametric BP_ND_ images for visual assessment of ^18^F-flutemetamol in a population with low amyloid burden improves interreader agreement. Implementing semiquantification in addition to visual assessment of SUVr images can reduce false-positive classification in this population.

## DISCLOSURE

This project received funding from the EU/EFPIA Innovative Medicines Initiative (IMI) Joint Undertaking (EMIF grant 115372) and the EU-EFPIA IMI-2 Joint Undertaking (grant 115952). This joint undertaking receives support from the European Union’s Horizon 2020 research and innovation program and EFPIA. Support was also received from the NIHR UCLH Biomedical Research Center, and in-kind sponsoring of the PET tracer was received from GE Healthcare. Philip Scheltens received grants from GE Healthcare, Piramal, and Merck, paid to his institution, and speaker’s fees paid to the Alzheimer Center, VU University Medical Center, Lilly, GE Healthcare, and Roche. Pieter Jelle Visser received research support from Biogen and grants from EU/EFPIA IMI Joint Undertaking, EU Joint Programme–Neurodegenerative Disease Research (JPND), ZonMw, and Bristol-Myers Squibb; served as a member of the advisory board of Roche Diagnostics; and received nonfinancial support from GE Healthcare. Frederik Barkhof received payment and honoraria from Bayer-Schering Pharma, Sanofi-Aventis, Genzyme, Biogen-Idec, TEVA, Merck-Serono, Novartis, Roche, Jansen Research, IXICO Ltd., GeNeuro, and Apitope Ltd. for consulting; payment from the Serono Symposia Foundation, IXICO Ltd., and MedScape for educational presentations; and research support via grants from EU/EFPIA Innovative Medicines Initiative Joint Undertaking (AMYPAD consortium), EuroPOND (H2020), U.K. MS Society, Dutch MS Society, PICTURE (IMDI-NWO), and ECTRIMS-MAGNIMS. No other potential conflict of interest relevant to this article was reported.
