# Supplementary material for: Assessing Amyloid Pathology in Cognitively Normal Subjects Using 18F-Flutemetamol PET: Comparing Visual Reads and Quantitative Methods
Source: J Nucl Med. 2019 Apr;60(4):541–7. doi: 10.2967/jnumed.118.211532 (PMC6448465; doi:10.2967/jnumed.118.211532)
Supplement: Supplementary file 1 [file jnm211532SupplementalData.pdf]

Supplemental Table 1

Supplementary Table 1. Regional Binding and SUVr Overestimation		
**Regional ROI**	**Binding (BP_{ND})**	**SUVr overestimation**
Frontal	.20 ± .14*	.18 ± .12*
Parietal	.14 ± .12	.17 ± .11†
Temporal	.14 ± .09	.15 ± .10

***Significantly higher than parietal and temporal ROI (p < .01).**

†Significantly higher than temporal ROI (p < .01).

Supplementary Figure 1. Negative/positive classification based on (semi-)quantitative measures. A total of 149 cases had a concordant visual read on both the SUVr and BP_{ND} image and were used in a ROC analysis in order to determine the dichotomous negative/positive cut-off value of the (semi-)quantitative measurements.

A) Boxplot displaying the separation of groups based on the majority visual read of SUVr images and the spread of semi-quantitative values within groups. **B**) ROC curve representing the sensitivity/specificity corresponding to the decision threshold (1.52) based on semi-quantitative values. **C**) Boxplot displaying the separation of groups based on the majority visual read of BP_{ND} images and the spread of quantitative values within groups. **D**) ROC curve representing the sensitivity/specificity corresponding to the decision threshold (0.26) based on quantitative values.

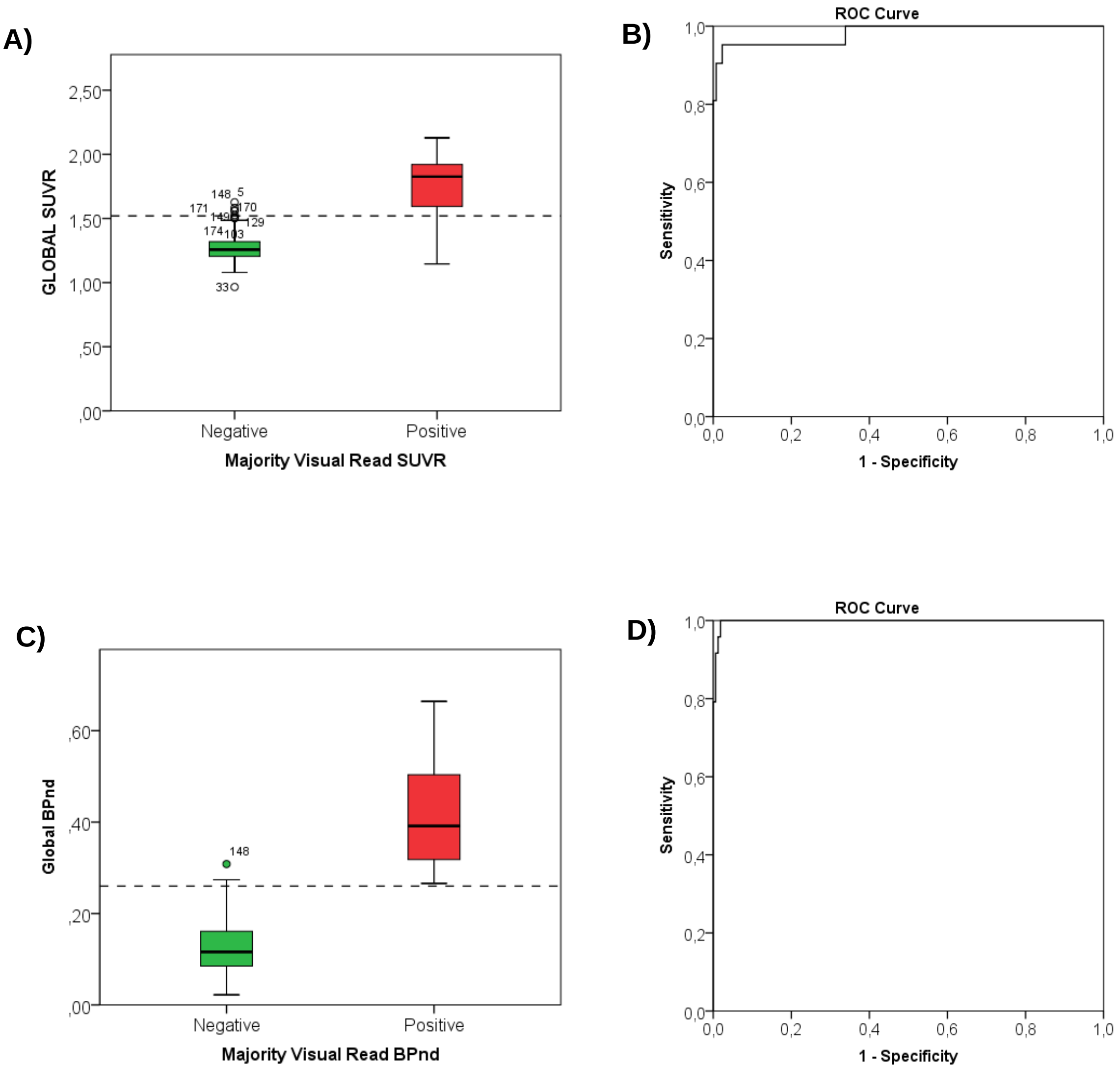

Supplemental Figure 2. Regional SUVr overestimations.
Diagrams showing the difference between **A**) frontal, **B**) parietal, and **C**) temporal SUVr overestimation and BP_{ND} for each subject with regard to visual read. The overestimation of SUVr is higher with increasing cortical binding in all regions.

A)

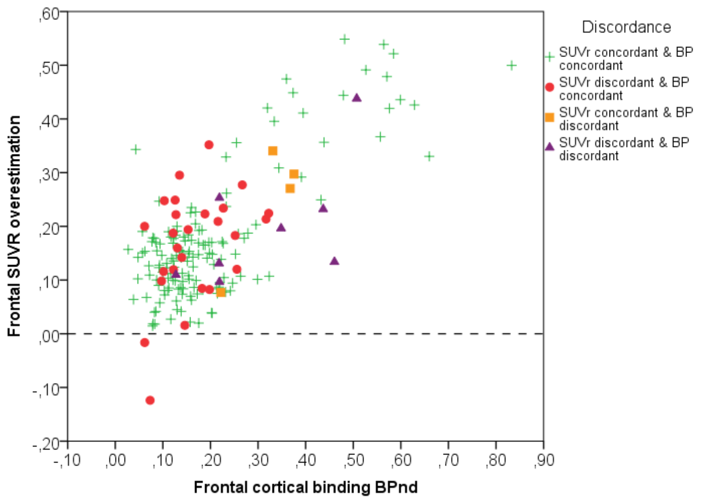

B)

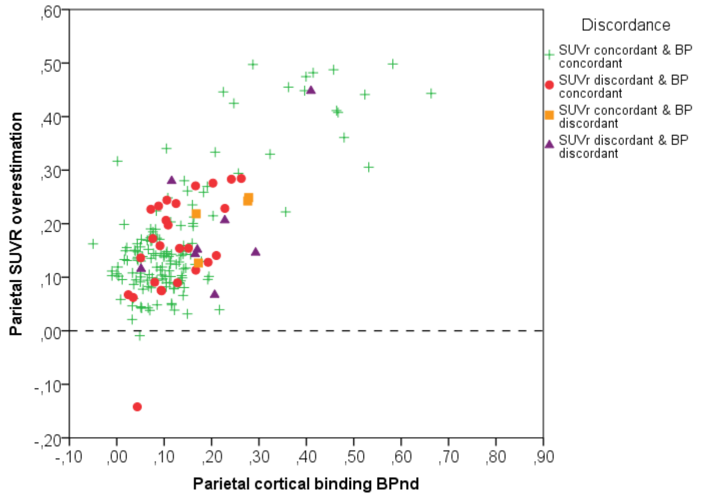

C)

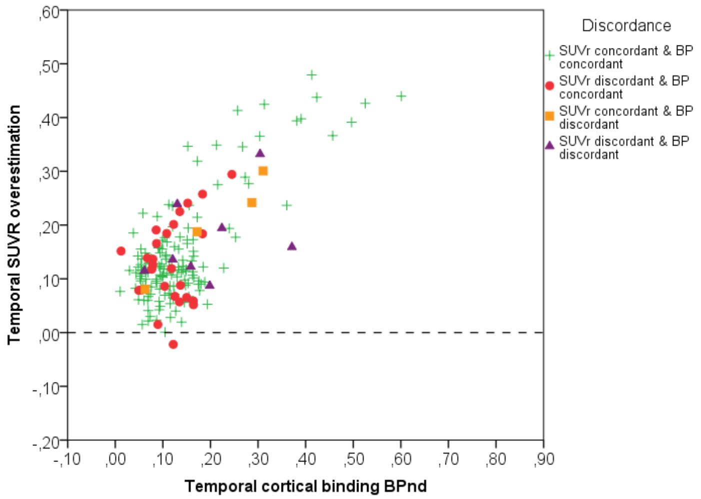
Discordance
SUVr concordant & BP concordant
SUVr discordant & BP concordant
SUVr concordant & BP discordant
SUVr discordant & BP discordant
Temporal SUVR overestimation
Temporal cortical binding BPnd
,60
,50
,40
,30
,20
,10
,00
-,10
-,20
-,10 ,00 ,10 ,20 ,30 ,40 ,50 ,60 ,70 ,80 ,90
